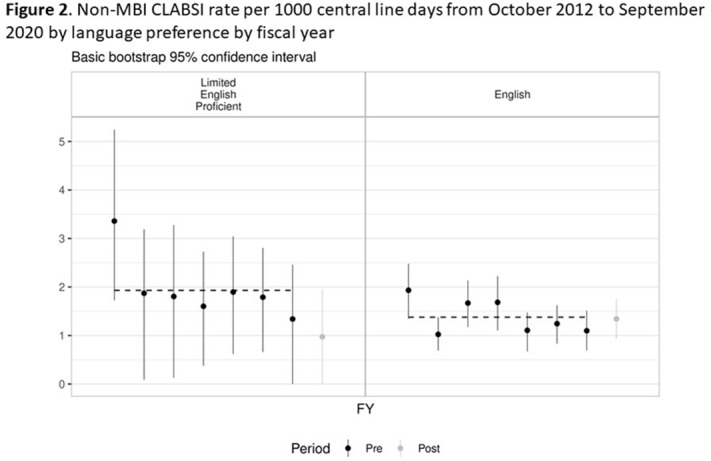# Inequities in CLABSI Rates in a Children’s Hospital by Race, Ethnicity, and Language Preference

**DOI:** 10.1017/ash.2021.80

**Published:** 2021-07-29

**Authors:** Caitlin McGrath, Matthew Kronman, Danielle Zerr, Brendan Bettinger, Tumaini Coker, Shaquita Bell

## Abstract

**Background:** Systemic racism results in health inequities based on patient race, ethnicity, and language preference. Whether these inequities exist in pediatric central-line–associated bloodstream infections (CLABSIs) is unknown. **Methods:** This retrospective cohort study included patients with central lines hospitalized from October 2012 to June 2019 at our tertiary-care children’s hospital. Self-reported race, ethnicity, language preference, demographic, and clinical factors were extracted from the electronic health record. The primary outcome was non–mucosal barrier injury (non-MBI) CLABSI episodes as defined by the NHSN. CLABSI rates between groups were compared using χ^2^ tests and Cox proportional hazard regression. We adjusted for care unit, age, immunosuppressed status, diapered status, central-line type, line insertion within 7 days, daily CLABSI maintenance bundle compliance, number of blood draws and IV medication doses, and need for total parental nutrition, extracorporeal membrane oxygenation, and renal replacement therapy. In mid-2019, we engaged stakeholders in each care unit to describe preliminary findings and to identify and address potential drivers of observed inequities. **Results:** We included 337 non-MBI CLABSI events over 230,699 central-line days (CLDs). The overall non-MBI CLABSI rate during the study period was 1.46 per 1,000 CLDs. Unadjusted CLABSI rates for black or African American (henceforth, “black”), Hispanic, non-Hispanic white, and Asian (the 4 largest race or ethnicity groups by CLDs) patients were 2.74, 1.53, 1.42, 1.24 per 1,000 CLDs, respectively (*P* < .001) (Table 1). Unadjusted CLABSI rates for patients with limited-English proficiency (LEP) and English-language preference were 1.98 and 1.38 per 1,000 CLDs, respectively (*P* = .014). After adjusting for covariates, the hazard ratio (HR) point estimate for CLABSI rate remained higher for black patients (HR, 1.50; 95% CI, 0.99–2.28) and patients with LEP (HR, 1.33; 95% CI, 0.87–2.05), compared to the reference group based on largest CLD. The differences in CLABSI rate by race or ethnicity and language were more pronounced in 2 of our 6 care units. Stakeholder engagement and analysis of hospital data revealed opportunities on those units for improved (1) interpreter utilization and (2) line maintenance observation practices by race/ethnicity and language preference (data not shown). These findings and CLABSI rates over time by race/ethnicity and language preference (Figures 1 and 2) were shared with frontline staff. **Conclusions:** In our children’s hospital, CLABSI rates differed based on patients’ self-reported race, ethnicity, and language preference, despite controlling for factors commonly associated with CLABSI. Identifying inequities in CLABSI rates and mitigating their determinants are both essential to the goal of achieving equitable care.

**Funding:** No

**Disclosures:** None

**Table 1.**
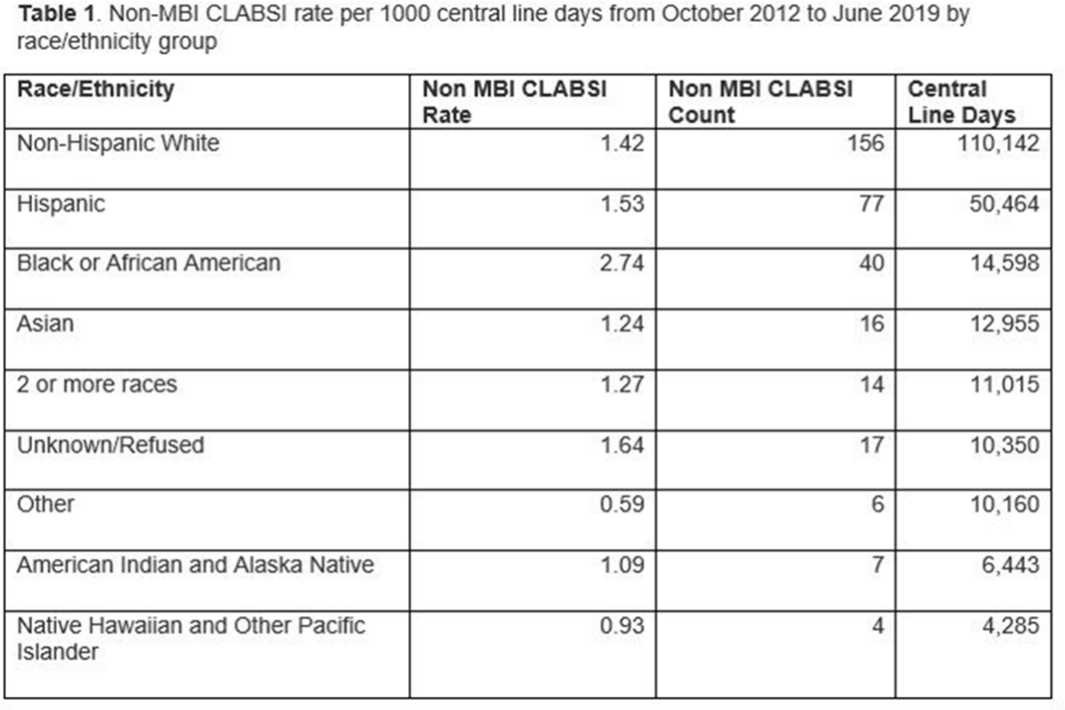

**Figure 1.**
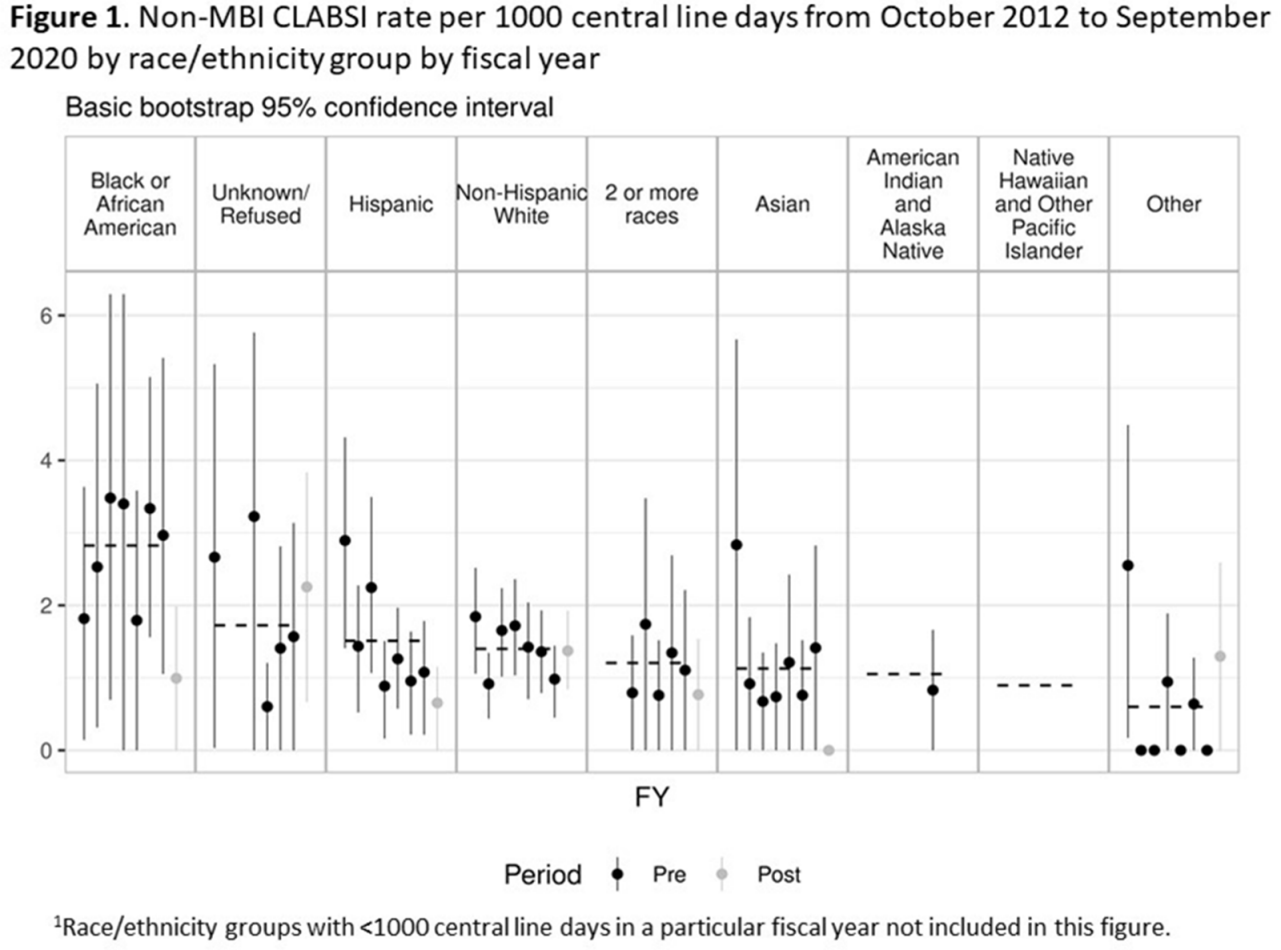

**Figure 2.**